# Influence of Amoxicillin Dosage and Time of Administration on Postoperative Complications After Impacted Third Molar Surgery: A Randomized, Double-Blind, Controlled Clinical Trial

**DOI:** 10.3390/antibiotics14121209

**Published:** 2025-12-01

**Authors:** Pablo Valverde-Martínez, Maria Victoria Olmedo-Gaya, Carlota Castro-Gaspar, Francisco Manuel Ocaña-Peinado, Francisco Javier Manzano-Moreno, Candela Reyes-Botella

**Affiliations:** 1 School of Dentistry, University of Granada, 18071 Granada, Spain; pablovmartinez@correo.ugr.es (P.V.-M.); mvolmedo@ugr.es (M.V.O.-G.); carlotacastrogaspar@gmail.com (C.C.-G.); creyes@ugr.es (C.R.-B.); 2 Department of Stomatology, School of Dentistry, University of Granada, 18071 Granada, Spain; 3 Department of Statistics and Operations Research, University of Granada, 18071 Granada, Spain; fmocan@ugr.es; 4 Biomedical Group (BIO277), University of Granada, 18071 Granada, Spain; 5 Instituto Investigación Biosanitaria, ibs.Granada, 18071 Granada, Spain

**Keywords:** amoxicillin, third molar, postoperative infection, antibiotic prophylaxis, RCT

## Abstract

**Background/objective:** The objective of this research was to evaluate how different antibiotic prophylaxis protocols influence the development of postoperative complications after the extraction of impacted mandibular third molars. **Methods:** This investigation was designed as a double-blind, randomized controlled trial. A total of eighty patients who fulfilled the inclusion criteria were randomly allocated to one of three treatment groups. Group 1 (*n* = 27) was administered 2 g of oral amoxicillin one hour prior to the procedure; Group 2 (*n* = 27) received 500 mg of oral amoxicillin every eight hours for five days following the surgery; and Group 3 (*n* = 26) was given 750 mg of oral amoxicillin every eight hours for the same postoperative period. The outcome variables measured were infectious complications, postoperative pain, postoperative inflammation, and others such as the rescue medication taken by the patient. **Results:** Two patients in each group showed signs of infection after the extraction of the impacted third molar (*p* = 0.412). No significant differences were observed in pain intensity between the different study groups, nor in the intensity of inflammation. No statistically significant differences were observed in the consumption or non-consumption of rescue analgesics. **Conclusions:** The results of the present study suggest that the preoperative administration of 2 g of amoxicillin in the extraction of impacted third molars in healthy patients could be sufficient to prevent infectious complications. The administration of postoperative doses of 500 mg every 8 h or 750 mg every 8 h does not generate benefits in patients in terms of postoperative pain or inflammation.

## 1. Introduction

The use of antibiotic prophylaxis in third molar surgery remains common practice, even though the scientific evidence supporting its effectiveness in healthy patients is still inconclusive [1,2,3,4]. Lower third molar extraction is considered Class II (clean-contaminated) surgery [5] and is one of the most frequent procedures in oral and maxillofacial surgery. Postoperative oral infections can lead to symptoms such as swelling, trismus, fever, pus formation, localized infections (e.g., alveolitis), and pain [6,7]. Although the incidence of infection is low (less than 5%) [8], the impact on quality of life in the postoperative period and its consequences in the workplace and economically are high [9]. Prophylactic treatment with antibiotics in healthy individuals is administered before or after extraction to prevent infectious complications [10]. However, the potential advantages of prophylactic antibiotic use must be carefully balanced against the well-documented risk of increasing bacterial resistance, which poses a major global public health concern [11,12].

The available literature does not provide a definitive consensus on whether antibiotic prophylaxis should or should not be used in impacted lower third molar extractions. This lack of agreement is likely due to several methodological limitations found in existing studies, including small sample sizes, inadequate experimental designs, poor group comparability, heterogeneity among study populations, and variations in antibiotic administration routes. Furthermore, some meta-analyses report results that contradict those of individual studies, likely due to the larger pooled sample sizes [1,13,14]. In a RCT previously published by our research group [15], we found that antibiotic prophylaxis significantly reduced the risk of surgical site infection (SSI) following lower third molar extraction. However, the antibiotic doses used in that study were higher than current recommendations. Consequently, we have designed a new RCT using lower antibiotic doses to minimize the adverse effects associated with antibiotic therapy.

Additionally, there is insufficient evidence regarding the most effective timing of prophylaxis—whether it should be administered preoperatively or postoperatively. Although dry socket (DS) is primarily an inflammatory reaction of the alveolar bone to surgical trauma, its association with bacterial contamination supports the use of antibiotics to prevent it [1]. For this reason, we are considering a second complementary study to clarify whether a single 2 g dose of amoxicillin may be sufficient to reduce the incidence of SSI and DS without significantly increasing microbial resistance.

Based on the literature, in which we observe how clinical trials continue to be published using different antibiotic administration regimens in retained third molar surgery, the objective of this RCT was to compare the efficacy of therapy with different antibiotic regimens (2 g of amoxicillin 1 h before surgery; 750 mg of amoxicillin every 8 h for 5 days after surgery; 500 mg of amoxicillin every 8 h after surgery) as a preventive measure against possible complications (infection, pain, and inflammation) of impacted mandibular third molar extraction.

## 2. Results

### Patient Characteristics and Interventions

**Figure 1:**
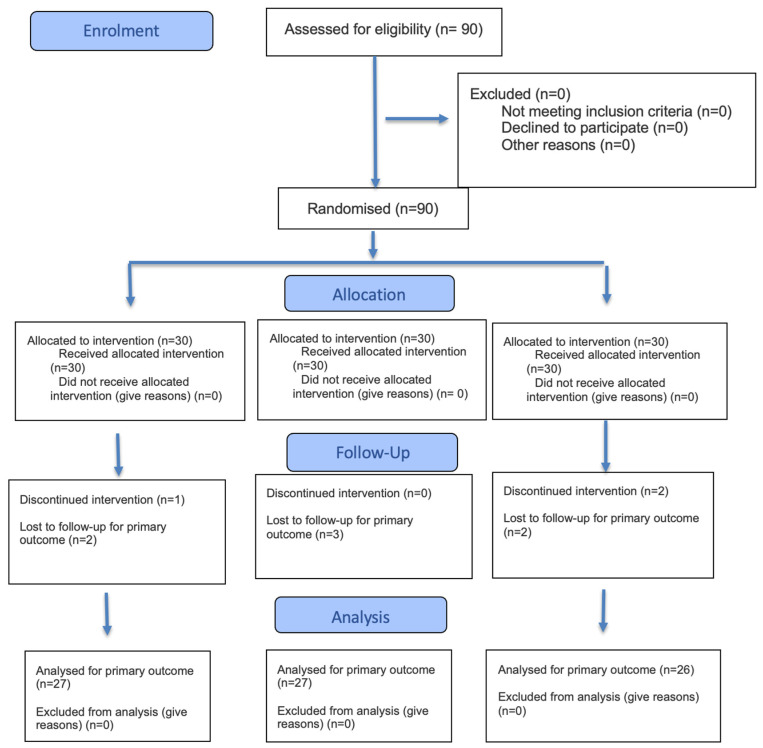
Flowchart of the enrollment process according to Consort Guideline.

We obtained a total sample of 80 patients, randomly divided into three groups: 27 patients in group 1, 27 patients in group 2, and 26 patients in group 3. Figure 1 depicts the flow of patients through the study.

The groups were homogeneous (no statistically significant differences were observed) for the predictive variables of gender, periodontal disease, tobacco use, difficulty according to Pedersen’s classification [16], odontosection, degree of osteotomy, duration of surgery, type of incision, periosteal tear, cause of extraction and number of sutures. A summary of these variables is shown in Table 1.

Table 2 shows data on the presence of infection and the use of rescue analgesics by patients. No statistically significant differences were observed between groups.

**Figure 2:**
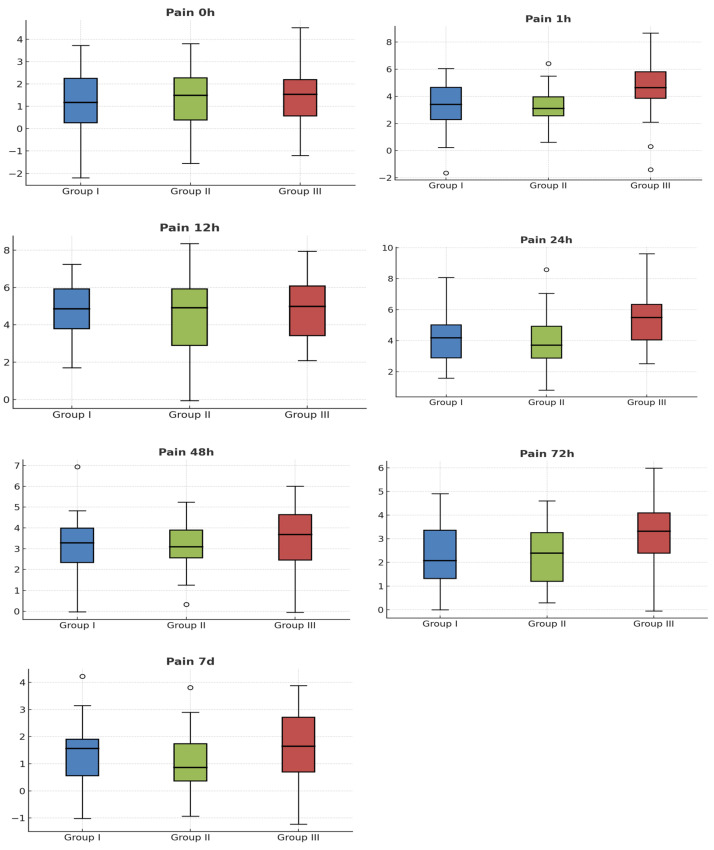
Box plots for comparison of pain scores (VAS) across different time points among the three groups. Circles are excentric values.

Figure 2 show the intensity of pain in the different study groups, with no significant differences between them. The average pain score 12 h after surgery was 4.17 in group 1, 4.63 in group 2, and 4.60 in group 3, measured on the VAS.

**Figure 3:**
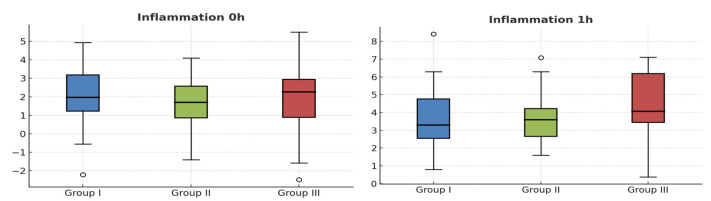

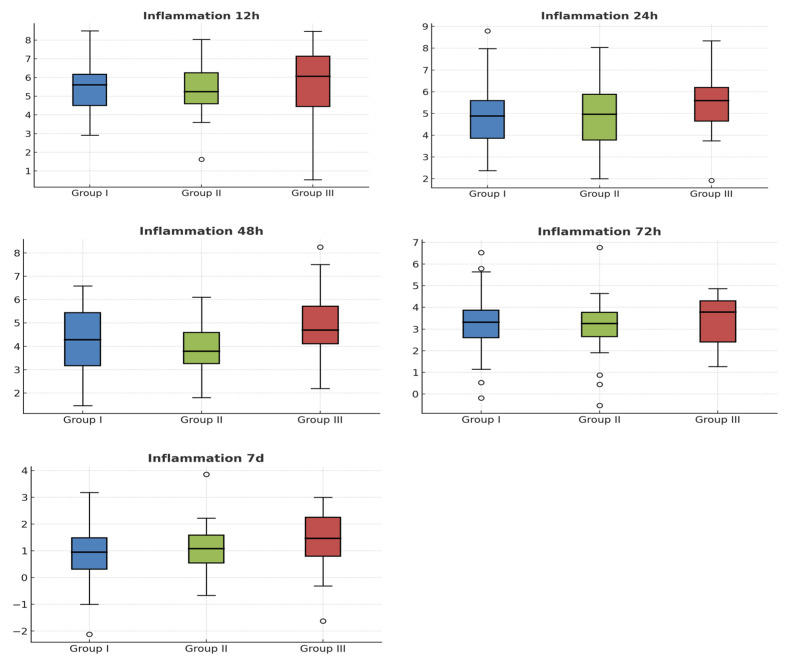
Box plots for comparison of inflammation scores (VAS) across different time points among the three groups. Circles are excentric values.

**Figure 4:**
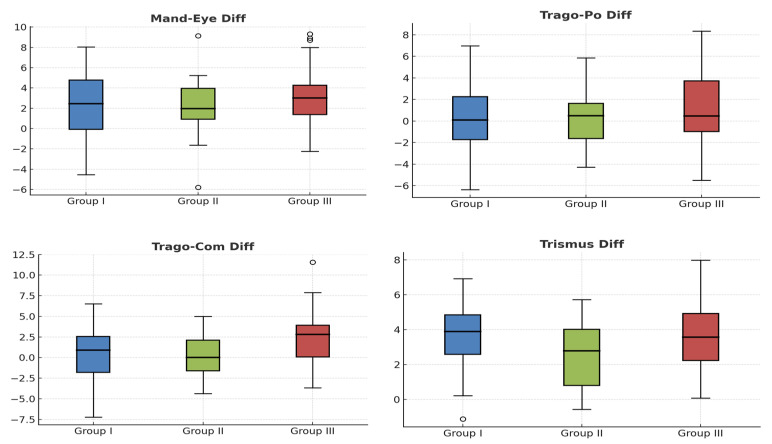
Box plots for comparison of inflammation scores with Gabka–Matsumura Craniometric Measurements. Circles are excentric values.

Figure 3 and Figure 4 show the inflammation data, both on the VAS and in the Gabka and Matsumura measurements, with an average score at 7 days of 1.70 in group 1, 1.71 in group 2 and 1.84 in group 3, measured on the VAS, with no statistically significant differences between the 3 groups.

## 3. Discussion

This RCT aimed to assess the impact of varying antibiotic doses and administration regimens on prophylaxis following the extraction of impacted mandibular third molars in healthy patients. The results showed no statistically significant differences among the three treatment groups. To reduce potential bias, factors related to the patient, the specific molar removed, and the surgical technique were considered, and group allocation was carefully controlled through randomization.

Bacterial contamination of the surgical site in the extraction of impacted third molars, which is considered a clean-contaminated surgery, is inevitable, either due to the bacterial flora of the patient or the environment [17]. The two infectious processes, such as DS and postoperative SSI, cause severe pain, functional impairment, and a decrease in quality of life [18].

The use of perioperative antibiotic prophylaxis can reduce the risk of postoperative infection and its characteristic symptoms and signs, as well as decrease the patient’s consumption of analgesics [19]. Although there are many references to this in the literature, there is still limited evidence regarding the most appropriate regimen to use, taking into account the increase in microbial resistance to the antibiotics used and the share of responsibility of dentists and oral surgeons in these figures [11,15,17,20].

In a previously published RCT conducted by our research team [15], we found that antibiotic prophylaxis markedly decreases the likelihood of developing a surgical site infection (SSI) following lower third molar removal. Moreover, patients who received a placebo before the procedure reported higher levels of pain and inflammation compared with those who were administered antibiotics either solely preoperatively or both pre- and postoperatively.

There are a number of studies that support the non-use of antibiotics prophylactically in the extraction of impacted third molars, in which variables such as those we have used in our trial are measured: pain, degree of inflammation and postoperative trismus. In some of them [21], central analgesics are used to alleviate pain, which is attributed to postoperative trauma. Kirnbauer et al. [22] reach the same conclusion, although in their study all patients were treated with 40 mg of methylprednisolone 1 h before the procedure. This may suggest that signs corresponding to postoperative inflammation are sometimes interpreted as signs of infection. Authors such as Torof et al. [4] and Isiordia-Espinoza et al. [23] conclude that routine use of antibiotics is not necessary.

The optimal dose and timing of antibiotic administration remain critical to maximize efficacy while minimizing the risks of resistance. In our study, different doses and timing of administration are compared, in agreement with several authors [1,24,25], to assess whether the efficacy of a single preoperative dose of amoxicillin is the same as when it is also used postoperatively, in a regimen similar to that used in the treatment of infection (500 mg or 750 mg every 8 h for 7 days).

The results obtained show no differences between Both the single preoperative dose and the combined pre- and postoperative regimens proved effective for the 500 mg and 750 mg formulations, aligning with the findings of Marcussen et al. [25]. These authors demonstrated that a single administration of 2 g of amoxicillin or 0.8 g of penicillin V effectively decreased the incidence of surgical site infections and alveolar osteitis, respectively. Similarly, Milic et al. [26] supported the use of a single preoperative 2 g dose of oral amoxicillin for lower third molar extractions, reporting a significant reduction in SSI rates. [1,27,28]. O-Camps Font et al. [1] and Wang et al. [28] also advocate preoperative doses of amoxicillin to prevent postoperative infection. Siddiqi et al. [24] measured infection parameters at 3, 7, and 14 days after administering a preoperative dose of 1 g of amoxicillin versus a regimen consisting of an initial preoperative dose of 1 g followed by 500 mg every 8 h for the next 2 days, obtaining differences between the two groups in terms of early infection risk, but not in the medium (7 days) or long (14 days) term. However, Falci et al. [29] found in a SR that Amoxicillin—particularly the 750 mg formulation and the combination of amoxicillin with clavulanic acid (500 mg + 125 mg)—administered postoperatively every eight hours for periods ranging from 3 to 7 days, has been shown to produce better infection-related outcomes than placebo [30]. This finding is unsurprising, as the same dosage used to treat active infections is also capable of preventing them. Nevertheless, the routine use of such regimens for prophylaxis after third molar surgery should be approached cautiously, given that the incidence of postoperative infection or dry socket is relatively low (1–5%) [8]. In addition, it is well established that the risk of developing antibiotic resistance increases with longer durations of antibiotic exposure [31]. Current consensus guidelines recommend that prophylactic antibiotics be administered within 24 h after the completion of surgery. Effective prophylaxis requires maintaining adequate concentrations of a suitable antimicrobial agent in the serum, tissues, and surgical site for the entire duration during which the incision remains open and vulnerable to bacterial contamination [32]. Taken together, these considerations support the use of single-dose preoperative protocols, which provide a balance of simplicity and effectiveness while potentially reducing the adverse effects associated with prolonged antibiotic use.

The recommendations published in recent meta-analyses are often not implemented in daily practice. This emphasizes the need to establish standardized guidelines to support clinical decision-making [33]. The irrational use of antibiotics can lead to an unjustified increase in economic costs and adverse reactions such as allergies, toxicity, gastrointestinal disorders and bacterial resistance [34,35]. Recent studies have demonstrated a direct relationship between antibiotic consumption and the emergence and spread of resistant bacterial strains [36].

We selected amoxicillin for this study, in common with many other authors [8,18] as it is the current antibiotic of choice for this type of surgery [9,21]. Antibiotics used in other studies have included amoxicillin/clavulanic acid [30], macrolides [37], nitroimidazoles [38], clindamycin [39], or even intravenous amoxicillin [40].

The main limitation of this study is the sample size, which although estimated according to the Wittes guidelines [41], the results should be interpreted with caution and more RCTs with larger sample sizes should be performed.

The researchers in this study have compared different doses and times of administration to help minimize the prescription of antibiotics in dental practice. This study provides clinically relevant information to professionals who perform impacted third molar extractions, with the aim of increasing their adherence to recommendations when prescribing prophylactic antibiotics and preventing their misuse. Further research is warranted on the need for and timing of antibiotic prophylaxis in patients undergoing impacted mandibular third molar extraction.

## 4. Materials and Methods

### 4.1. Study Design, Patient Selection and Sample Size

This single-centre, double-blind randomized clinical trial was carried out in patients scheduled for the surgical removal of impacted mandibular third molars at the Clinic of the School of Dentistry, University of Granada (Spain), between January 2024 and May 2025. Exclusion criteria included being under 18 years of age; pregnancy or lactation; the presence of systemic disease (only ASA Class I patients were eligible); known hypersensitivity to the study medication or related antibiotics (such as penicillins); antibiotic use within the week preceding surgery; and the presence of pericoronitis or apical radiolucency associated with the tooth indicated for extraction.

All subjects provided written informed consent prior to participation. The study was conducted in accordance with the ethical principles established in the Declaration of Helsinki. Approval for the protocol was granted by the Human Research Ethics Committee of the University of Granada (reference: 1066/CEIH/2020), and the trial was registered in the Australia and New Zealand Clinical Trials Registry (ANZCTR; ACTRN12624001414505). Reporting followed the guidelines of the CONSORT 2025 statement for randomized clinical trials [42].

The main outcome assessed in this study was the occurrence of postoperative infection and the requirement for rescue antibiotic therapy. Drawing on data from prior research, a 2-point difference in these parameters was deemed clinically significant. The calculation assumed a 95% confidence interval, 90% statistical power, and a common standard deviation of 2.5, corresponding to one-quarter of the scale range described by Martin-Ares et al. [17], with an equal (1:1) allocation ratio between groups. Sample size estimation was performed using the Sample Sizer Release software (3.2.26 version, Microsoft Office Excel 2011; Microsoft Corp., Redmond, WA, USA), considering an alpha level of 0.05, 80% power, and an anticipated 15% attrition rate. Based on these parameters, the required sample size was determined to be 30 participants per group.

Of these, 10 were excluded due to intestinal intolerance to the antibiotic, incorrect use of the study medication or failure to attend the scheduled follow-up visits. The final sample included 80 participants (35 men and 45 women) between 18 and 63 years of age. Patients were enrolled consecutively and randomly allocated to one of the three study groups using a computer-generated randomization sequence. Each participant was assigned a random code (1, 2 or 3), with code 1 representing the amoxicillin (Laboratorios Cinfa SA, Pamplona, Navarra, Spain) 2 g group (1 h before surgery); Code 2 representing the amoxicillin 500 mg group (500 mg of oral amoxicillin every 8 h for 5 days post-surgery); or Code 3 representing the 750 mg group (750 mg of oral amoxicillin every 8 h for 5 days post-surgery).

All data were gathered by the principal investigator (PVM), who remained blinded to the patients’ group allocation. Both the participants and the surgeon were likewise unaware of the specific treatment each patient received. In addition, the statistics consultant did not have access to group assignments until the study had been fully completed.

### 4.2. Surgical Protocol

The surgeon and assistant carried out a surgical hand scrub, washing their hands and forearms up to the elbows with an antiseptic solution—either 4% chlorhexidine or 7.5% povidone-iodine—for approximately five minutes. They subsequently put on sterile gowns and gloves. Patients were fully draped with sterile covers, and the lips as well as the surrounding perioral skin were disinfected with a 10% povidone–iodine solution (Corsodyl, SmithKline Beecham, Brentford, UK). Immediately prior to the intervention, each patient completed a two-minute mouth rinse using 10 mL of 0.12% chlorhexidine solution (Perio-Aid, Dentaid SL, Barcelona, Spain), which was delivered through sterile syringes.

All surgical procedures were performed under local anesthesia with 4% articaine combined with epinephrine at a 1:100,000 ratio. (Ultracain, Normon SA, Madrid, Spain). The extractions were performed by postgraduate students in the final year of their master’s degree, under the close supervision of an experienced oral surgeon (CRB).

Depending on the radiographic assessment of the surgical difficulty, either a scalloped or a releasing incision was made, followed by elevation of a full-thickness mucoperiosteal flap to expose the tooth and surrounding bone. Osteotomy was carried out using a handpiece equipped with a round tungsten carbide bur, and when necessary, odontosection was completed with a turbine and fissure bur. After tooth removal, the socket was sutured with 3.0 silk (Normon SA, Madrid, Spain) and compressed for 30 min using gauze impregnated with 0.20% chlorhexidine gel (Lacer SA, Barcelona, Spain).

Postoperative instructions included rinsing with 0.12% chlorhexidine mouthwash (Perio-Aid; Dentaid SL, Barcelona, Spain) once daily after toothbrushing for one week. Patients were scheduled for suture removal at the one-week follow-up visit.

All participants received 400 mg of ibuprofen every eight hours for four days following surgery. In cases where pain control was insufficient after one hour, 1 g of paracetamol was recommended as a rescue analgesic.

If postoperative infection was suspected or evident, patients were prescribed a rescue antibiotic consisting of one tablet of amoxicillin (875 mg) combined with clavulanic acid (125 mg) every eight hours for seven days.

### 4.3. Study Variables

The study variables were divided into predictor variables and outcome variables. Predictor variables were additionally classified according to whether they were associated with the patient, the tooth, or the surgical procedure. All information was recorded and evaluated by the principal investigator. Patient-related factors included sex, age, level of oral hygiene (good or poor), presence of periodontal disease (yes or no), bruxism (yes or no), and smoking status (yes or no).

For the third molar, the variables considered were the numerical score obtained using the Pedersen index (18) and the indication for extraction, which could be preventive, orthodontic, or due to pathology affecting the adjacent tooth.

The surgical variables were the duration of surgery (in minutes); degree of osteotomy (none, mesial-vestibular, mesial-distal-vestibular, mesial-distal-vestibular-lingual/occlusal); coronal section (yes/no); and number of sutures.

The primary outcome variables of this study were the presence of postoperative infection and the requirement for rescue antibiotic treatment. Secondary outcomes were assessed through variables related to inflammation and pain. Baseline facial inflammation was recorded immediately before surgery using the method described by Gabka and Matsumura [43], measuring the distance from the mandibular angle to the lateral canthus, from the tragus to the pogonion, and from the tragus to the corner of the mouth.

During the postoperative period (7 days after the extraction), each participant filled out a data collection form that included the following outcome measures: pain intensity recorded at the time of surgery and at 1, 12, 24, 48, and 72 h, as well as at 7 days postoperatively, using a horizontal visual analogue scale (VAS) ranging from “no pain” to “worst pain imaginable”; postoperative swelling at the surgical site assessed with a similar VAS anchored by “no swelling” and “worst swelling imaginable”; intake of 1 g paracetamol as rescue analgesia (yes/no); need for rescue antibiotic therapy (yes/no), as previously defined by the principal investigator; and any adverse effects (such as nausea, vomiting, drowsiness, motion sickness, tremors, sweating, dyspepsia, diarrhea, bleeding, or dizziness). Additionally, patients rated their overall perception of the prescribed medication on a four-point scale (1 = poor, 2 = average, 3 = good, 4 = very good).

Patients returned to the clinic 7 days postoperatively for suture removal and to submit the completed questionnaire. During this follow-up visit, the principal investigator measured postoperative swelling (in millimetres) using the same Gabka and Matsumura technique and recorded the presence or absence of infection or dry socket at the extraction site. Infection was diagnosed clinically based on one or more of the following criteria: detection of an abscess by fluctuation or purulent drainage; a body temperature above 37.8 °C lasting more than 24 h without another identifiable cause; or severe pain and/or swelling persisting for at least 48 h after surgery, without an alternative explanation and showing improvement with antibiotic therapy.

### 4.4. Statistical Analysis

Statistical analysis was performed using SPSS v 30.0 (IBM Corp, Armonk, NY, USA, 2024). This analysis was carried out in two stages: first, a descriptive analysis was performed, and second, a statistical inference analysis was performed. In the descriptive summary, the qualitative variables were described using frequency and contingency tables. For the quantitative variables, a statistical summary was performed using the mean, standard deviation, median and interquartile range (IQR). The normality of the variables was checked using the Shapiro–Wilk test. In the statistical inference analysis, to compare the quantitative variables in the case of several samples, a one-way analysis of variance (ANOVA) was carried out in the presence of normality, or, conversely, the Kruskall–Wallis test in the absence of normality. The homogeneity between samples in all cases, 2 or more samples, was tested using the Levene test. Qualitative variables were analyzed using the chi-square test with Fisher’s correction for 2 × 2 tables. The Bonferroni test, the Games-Howell test (as appropriate based on the Levene test) or the Mann–Whitney test (in case of non-normality) were the procedures used for pairwise multiple comparisons. In all tests performed, the significance level, α, was set at α = 0.05.

## 5. Conclusions

The results of the present study suggest that the preoperative administration of 2 g of amoxicillin in the extraction of impacted third molars in healthy patients could be sufficient to prevent infectious complications.

## Figures and Tables

**Table 1:** Demographic, clinical, and extraction characteristics and tooth-specific outcomes by Study Group.

Characteristic	Group 1 (*n* = 27)	Group 2 (*n* = 27)	Group 3 (*n* = 26)	*p*-Value
Age (years)				0.245
Mean ± SD	25.2 ± 4.1	24.8 ± 3.9	26.3 ± 4.5	
Median	25.0	24.5	26.0	
IQR	[22.0–28.0]	[22.0–27.0]	[23.0–29.0]	
Sex (M/F)	16/14	13/11	14/12	0.991
Oral Hygiene (1–2)				0.834
Mean ± SD	1.2 ± 0.4	1.1 ± 0.3	1.3 ± 0.5	
Median	1.0	1.0	1.0	
IQR	[1.0–1.0]	[1.0–1.0]	[1.0–1.0]	
Smoking Status	3 (10.0%)	2 (8.3%)	4 (15.4%)	0.721
Presence of EP	4 (13.3%)	3 (12.5%)	5 (19.2%)	0.689
Pedersen Scale				0.067
Mean ± SD	5.4 ± 1.2	5.3 ± 1.1	5.9 ± 1.3	
Median	5.0	5.0	6.0	
IQR	[5.0–6.0]	[5.0–6.0]	[5.0–7.0]	
**Tooth Position**				0.218
38	16 (60%)	11 (40%)	13 (50%)	
48	11 (40%)	16 (60%)	13 (50%)	
**Angulation (Winter’s)**				0.032 *
Mesioangular	12 (44.4%)	6 (22.2%)	8 (30.8%)	
Horizontal	8 (29.6%)	11 (40.74%)	7 (26.9%)	
Vertical	7 (25.92%)	10 (37.03%)	11 (42.3%)	
**Pell & Gregory Class**				0.175
IIB	15 (55.5%)	12 (44.4%)	16 (61.5%)	
IIIA	12 (44.4%)	15 (55.5%)	10 (38.5%)	
Operative Time (min)				0.012 *
Mean ± SD	36.8 ± 12.4	34.2 ± 11.8	46.5 ± 13.2	
Median	35.0	32.5	45.0	
IQR	[25.0–45.0]	[22.5–42.5]	[35.0–55.0]	
Incision Type (1–2)				0.067
Mean ± SD	1.4 ± 0.5	1.2 ± 0.4	1.1 ± 0.3	
Median	1.0	1.0	1.0	
IQR	[1.0–2.0]	[1.0–1.0]	[1.0–1.0]	
Osteotomy				0.234
Mean ± SD	2.1 ± 1.0	1.9 ± 0.9	2.4 ± 1.1	
Median	2.0	2.0	3.0	
IQR	[2.0–3.0]	[2.0–2.0]	[2.0–3.0]	
Odontosection	8 (26.7%)	6 (25.0%)	9 (34.6%)	0.678
Number of Sutures				0.156
Mean ± SD	3.8 ± 1.1	3.7 ± 1.0	3.4 ± 0.9	
Median	4.0	4.0	3.0	
IQR	[3.0–4.0]	[3.0–4.0]	[3.0–4.0]	
Periosteal Tear	2 (6.7%)	1 (4.2%)	3 (11.5%)	0.589

* *p*-value one-way analysis of variance (ANOVA) or Kruskall–Wallis.

**Table 2:** Postoperative Infection and Rescue Rates by Treatment Group.

Group	No Infection [*n* (%)]	Infection [*n* (%)]	No Rescue [*n* (%)]	Rescue [*n* (%)]
**1 (*n* = 27)**	25 (92.5)	2 (7.5)	28 (93.3)	2 (6.7)
**2 (*n* = 27)**	25 (92.5)	2 (7.5)	22 (91.7)	2 (8.3)
**3 (*n* = 26)**	24 (92.3)	2 (7.7)	22 (84.6)	4 (15.4)
***p*-value**	0.412		0.287	

## Data Availability

The datasets used and/or analyzed during the current study are available from the corresponding author on reasonable request.
